# Scoping review of studies on food marketing in Latin America: Summary of existing evidence and research gaps

**DOI:** 10.11606/S1518-8787.2019053001184

**Published:** 2019-12-20

**Authors:** Maria Manuela Chemas-Velez, Luis F. Gómez, Alcides Velasquez, Mercedes Mora-Plazas, Diana C. Parra

**Affiliations:** I Pontificia Universidad Javeriana Facultad de Medicina Departamento de Medicina Preventiva y Social Bogotá Colombia Pontificia Universidad Javeriana. Facultad de Medicina. Departamento de Medicina Preventiva y Social. Bogotá, Colombia.; II Universidad Nacional de Colombia Facultad de Medicina Departamento de Nutrición Humana Bogotá Colombia Universidad Nacional de Colombia. Facultad de Medicina. Departamento de Nutrición Humana. Bogotá, Colombia.; III Washington University in St. Louis School of Medicine Program of Physical Therapy St. Louis USA Washington University in St. Louis. School of Medicine. Program of Physical Therapy. St. Louis, USA.; IV University of Kansas Department of Communication Studies LawrenceKS USA University of Kansas. Department of Communication Studies. Lawrence, KS, USA.

## Abstract

**OBJECTIVE:**

To document the evidence about marketing of ultra-processed foods and “non-alcoholic” beverages in Latin America.

**METHODS:**

We performed a structured search of quantitative and qualitative studies in PubMed, SciELO and LILACS, published between January 2000 and May 2017 and conducted in Latin America. We conducted a quality assessment following a standardized tool and a thematic analysis to identify key typologies of marketing across studies

**RESULTS:**

Out of 521 studies screened by title and abstract, we included 36 in this review; 27 of them analyzed television advertisement. Other marketing channels studied were food packaging, point of sale and outdoor advertisement. Studies found television advertises foods and beverages that are mostly ultraprocessed foods and have low nutritional value, particularly those promoted during children’s programming. We also observed children have a literal interpretation of images printed on food packaging, so this can be deceiving. Several studies also found proximity to unhealthy foods may increase their consumption. Finally, the thematic analysis identified the following typologies of food marketing: a) television advertisement, b) food packaging marketing, c) marketing strategies at points of sale and d) other marketing strategies. We found almost no advertisements for unprocessed or minimally processed foods such as fruits and vegetables. We did not find any studies on digital marketing conducted in the region.

**CONCLUSIONS:**

This review found that the main channel of food marketing was television advertising. This synthesis provides insights to the challenges unhealthy eating represents to the public health of Latin America and identifies knowledge gaps to guide future research.

## INTRODUCTION

According to the Global Burden of Disease Study, unhealthy dietary patterns have become the third risk factor associated with disability-adjusted life years and the second risk factor associated with mortality in Latin America and Caribbean^1^. Evidence shows the consumption of ultra-processed foods, including sweetened beverages, is associated with obesity and type 2 diabetes^2^. Changes in eating patterns and the growing consumption of ultra-processed foods and beverages^5^ characterize Latin America’s rapid nutrition transition_._ For instance, a study conducted in Brazil showed an increase of 4.8% in the energy value of ultra-processed foods between 2002-2003 and 2008-2009^6^. Another Mexican study found the calories from beverages frequently marketed to children increased from 161 kcal in 1999 to 310 kcal in 2006^7^.

Marketing plays an important role in this transition process as it allows to modify and reinforce social norms that dictate the type of foods to be eaten and the manner and time to eat them. Marketing often depicts foods being consumed in situations other than mealtimes, away from the table and in unlimited quantities^8,9^. The food and beverage industries spend billions of dollars every year on advertising^10^, and the vast majority of promoted products are energy-dense and nutrient-poor^11^.

Children are extremely vulnerable to food marketing. They are highly impressionable, cannot recognize advertising intent, lack nutritional knowledge, and are motivated by immediate gratifications.^12,15,16^. Moreover, food companies target children for they have a strong influence in what families buy and establish brand loyalty at an early age, which has long-term effects on eating preferences and behaviors.^9,11,16^. For example, the risk of becoming an overweight adult is twice as much for an overweight child compared with a normal weight child^17^.

Several studies on food and beverage marketing have been conducted in Latin America; however, no recent syntheses summarize the main results from the available literature. Therefore, we performed a scoping review^19^ of studies that analyzed marketing of food and nonalcoholic beverages conducted in Latin America, in terms of exposure, marketing strategy, and nutritional value, using both qualitative and quantitative methods.

## METHODS

We conducted a scoping review following the Joanna Briggs Institute guidelines^18^, which is the criteria recommended by Colquhoun et al.^19^ and used PRISMA guidelines to design the protocol and report the results^20^. We documented the study objectives, methods, inclusion criteria and quality assessment in a study protocol. This form of knowledge synthesis enabled systematically searching, selecting, and synthesizing the existing evidence in this area of study^19^. The research questions were: What is the evidence regarding the marketing of food and nonalcoholic beverages conducted in Latin America? And, what are the key typologies of marketing across studies?

### Data Sources and Search Strategy

We took the evidence documented in this scoping review from original quantitative and qualitative studies in the area of food and beverage marketing, which were conducted in Latin and Central American countries and the Greater Antilles (Cuba, Hispaniola; Haiti and the Dominican Republic, Puerto Rico and Jamaica). In this review, marketing was assumed as *“*any form of commercial communication or message that is designed to, or has the effect of, increasee the recognition, appeal and/or consumption of particular products and services. It comprises anything that acts to advertise or otherwise promote a product or service” (WHO, p. 9)^21^.

After clearly identifying the research question, scoping reviews need to identify relevant studies. To ensure the comprehensiveness and range of our search, we investigated studies in the following electronic databases: PubMed, SciELO and LILACS. To further ensure the desired level of breadth, we included studies published in Spanish, Portuguese, French and English. The time frame of publication was from January 2000 to May 2017. The search included the following free terms, using the Boolean operator “OR”: “marketing”, “advertisement”, “publicity”, “promotion”, and “ad”. Moreover, the terms “food” and “beverage” were added to the query using the operator “AND” (Appendix I). This search strategy was defined to support sensitivity and reduce the risk of omitting relevant studies. In addition, we conducted a manual search to include relevant articles cited in the studies found, following recommendations of scoping reviews experts^19^. Finally, we contacted Latin American researchers and public health advocates members of the *Coalición Latinoamérica Saludable* (Healthy Latin America Coalition), to verify gray literature sources. We carried out the search until May 31, 2017.

### Study Selection and Inclusion Criteria

**Figure 1:**
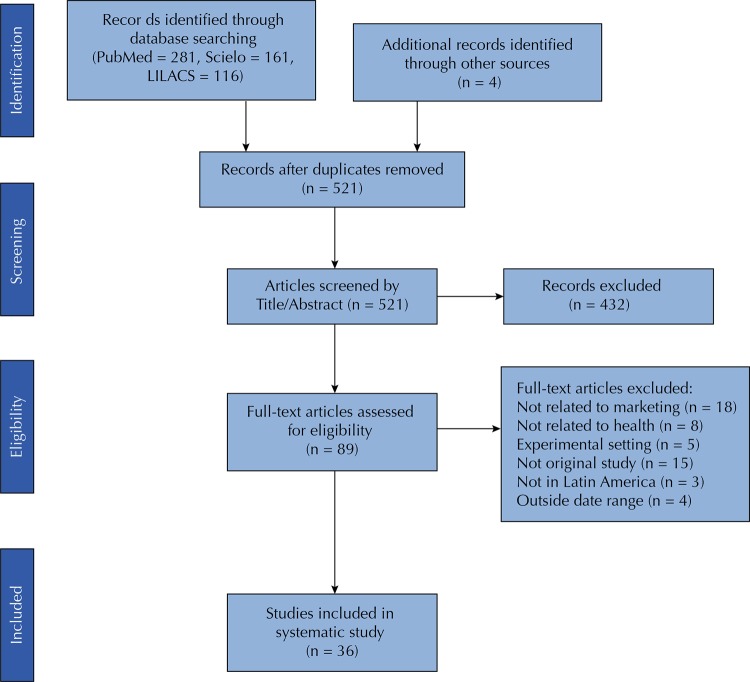
Study selection

Two of the authors undertook the study selection process, which can be seen in the PRISMA flow diagram (Figure 1) based on the content of titles and abstracts and in a second step based on the review of the manuscript text. In case of discrepancies, a third author reviewed the manuscript, and an agreement was obtained using a deliberative process. Studies that were not directly connected with health, nutrition and or food marketing were not included in this review.

### Data Extraction and Quality Assessment

For each study inclusion, two authors independently extracted the data, based on titles and abstracts. Subsequently, these two reviewers extracted the basic information from the manuscript texts and assessed the quality of each study. In case of disagreement, a third author was consulted. The following information was obtained and summarized from each study: authors, publication date, country, year in which the study was conducted, study design and objectives, study population, sample size, prevalence, and sociodemographic correlates.

A quality assessment was performed on both quantitative and qualitative articles following a standardized tool that has been used by authors in similar situations and that enables the application to several research methods^22^. Five items were assessed: a) Is there a clear and focused research question? b) Did the authors establish a theoretical framework? c) Was the methodology used appropriate for the objectives of the study? d) Did the authors recognize and describe the limitations of their study? and e) Did the authors clearly describe their results? The studies were classified for each of these criteria as “meeting the criteria”; 20 points, “partially meeting the criteria”; 10 points, and “not meeting the criteria”; 0 points. Finally, a percentage of criteria met were calculated and studies were labeled as being of poor quality (< 60%), fair quality (60%–90%), and good quality (> 90%).

### Analytical Approach

Two methods of analysis were used. Firstly, we briefly described the objective, study design, setting and main results of the studies. The second was a thematic analysis that was used to identify key typologies of marketing across studies^23^. The authors reviewed, refined and named these typologies during several meetings, considering the terminology proposed by the World Health Organization^22^. Since some studies addressed several topics of marketing, their findings might be included in several typologies.

## RESULTS

We identified 558 studies in our database search in PubMed, Scielo and LILACS and four additional studies were found by a manual search of cited articles and by contact with public health researchers, for a total of 562 studies. After adjusting for duplicates, 521 studies remained. Out of these, 432 were discarded based on title and abstract and 89 studies were selected for full text assessment. Among these, 53 articles did not meet the inclusion criteria and were discarded; 36 studies were included (Figure 1).

The Table 1 shows a general description of the main characteristics and findings of the 36 studies included in the review^24-59^. Ten studies were conducted in Brazil, eight in México, and five in Guatemala. The other studies were from Peru (n = 4), Argentina (n = 3), Chile (n = 3), Honduras (n = 1), El Salvador (n = 1), and Colombia (n = 1). In total, 27 studies were quantitative, six were qualitative and three used mixed method approaches.

**Table 1:** Studies conducted in Latin-American countries about food marketing and health.

Author and year of publication	Country and year of study	Study design and objective	Setting	Main results	Quality assessment
Sousa et al.^24^ (2002)	Brazil, 1998–2000	Quantitative study (cross-sectional). To analyze the quantity and quality of food advertised on the three main broadcast channels in Brazil	Three main broadcast channels were recorded during different times of the day on weekdays and Saturdays.	Food products were the most frequently advertised product with 27.4%. The category that appeared most often was fats, oils, sugars, and sweets (57.8%), followed by breads, cereals, rice, and pasta (21.2%), dairy products (11.7%), and lastly meat, eggs, and legumes (9.3%).	Fair quality
Olivares et al.^25^ (2003)	Chile, 2000	Quantitative study (cross-sectional). To determine attitudes towards television food advertising and eating behaviors among schoolchildren in different municipalities.	The researchers interviewed 271 schoolchildren (5th to 8th grade) from 3 different Chilean cities.	Almost all children (92%) reported watching television daily. When asked about television food commercials, 69.7% said they enjoyed them and 88.7% could remember at least one that they liked.	Fair quality
Fiates et al.^26^ (2008)	Brazil, 2005	Qualitative study. To conduct a qualitative assessment of the food choices, TV watching habits and physical activity habits among Brazilian children.	Twelve focus groups were conducted with primary private school students (7–10 years old, n = 57).	Most of the participants had a positive perception regarding the consumption of fruits and vegetables. Most also reported liking snack food and buying these products with their own money. Frequent intake of snack foods was not a habit for most students. Most of the students reported watching television daily.	Good quality
Ramírez-Ley et al.^27^ (2009)	Mexico, 2008	Quantitative study (cross-sectional). To assess television food commercials aired on both local and national public access channels and to evaluate their frequency, type, and audience.	Two free-access local channels and three free access national channels were recorded during weekday afternoons and Saturday mornings.	Food-related advertisements accounted for 22% of recorded commercials. Among these, 50% were child-oriented. The most frequently advertised foods were potato and corn chips (97%), desserts and cakes (89%), juices (77%) and sweetened cereals (75%). High-energy dense foods were disproportionately advertised to children (60%) when compared with those to adults (40%). Only 1% of all food advertisements promoted vegetables and fruits.	Good quality
Pérez-Salgado et al.^28^ (2010)	Mexico, 2007	Quantitative study (cross-sectional). To study the content, nutritional value and publicity strategy of food advertisements aired on free-access channels in Mexico City.	All public-access channels in Mexico City were recorded for one weekday and one day during the weekend.	Food products composed 17.6% of advertisements. Children programs had more advertisements when compared with general audience programs (19.89% vs. 15.46%), more food advertisements (25.8% vs. 15.4%) and higher frequency of advertisements for sweetened beverages (34.5% vs. 26.3%), and sweetened cereals (29.1% vs. 13.7%).	Good quality
Crovetto et al^29^ (2011)	Chile, 2010	Quantitative study (cross-sectional). To observe, register and identify television advertisements from different television channels that are part of the Chilean National Television Association and to describe the nutritional quality of the food products advertised.	Recordings were made of seven public access channels in Chile and advertisements were extracted and analyzed according to their frequency and duration. The foods advertised were classified as healthy, moderately healthy, and unhealthy based on the “Traffic Light System” by the UK Food Standard Agency.	A total of 16% of ads were identified as food advertisements. From these, 64% were related to unhealthy food, 27% to moderately healthy food and 9% to healthy food. The most frequently advertised food product was soft drinks (20%).	Fair quality
Olivares et al.^30^ (2011)	Chile, 2010	Quantitative study (cross-sectional). To determine the attitude of Chilean school children from different socioeconomic levels and different regions of the country towards marketing of foods and beverages.	Children from three different cities in Chile and from different socioeconomic status were selected. A previously validated questionnaire was used.	Between 26% and 66% of children reported liking food advertisements on television. In upper middle-income children, the percentage was similar between all three regions of the country (26% in Santiago, 29% in Chillán and 35% in Arica). In lower middle-income children, a marked increase was observed in Santiago, where 66% reported liking television foods commercials compared with 34% in Chillan and 26% in Arica. When researchers asked children if they remembered food commercials that they liked 66% said they did and 65% reported interest in trying new foods advertised by this medium. The food and beverage commercials that the children preferred were: sweetened beverages, fruit juices, chocolates, ice cream, breakfast cereals and potato chips.	Fair quality
Santos et al.^31^ (2012)	Brazil, 2008	Quantitative study (cross-sectional). To analyze the quantity and nutritional value of food items promoted on the Brazilian free-to-air television.	Two public access channels with the highest ratings in Brazil were recorded during two nonconsecutive weeks. Food advertisements were classified according to the food pyramid and nutritional values were obtained from the packaging.	Out of the food items, advertised 85% were sugars, oils, or fats. No advertisements were found for fruits or vegetables.	Fair quality
Rodrigues et al^32^ (2012)	Brazil, 2006 and 2008	Qualitative study. To compare eating habits and consumer behaviors of children from different socioeconomic levels and to investigate their relation to television watching habits.	Twenty-three focus groups were conducted in 111 students between 7 and 10 years old from private and public schools. The households of private school students had higher income than those of public school students.	Most children had pocket money they could spend. Most reported liking and watching television daily. Most children said they liked and wished to buy food products advertised on television.	Good quality
Costa et al.^33^ (2012)	Brazil, 2009	Quantitative study (cross-sectional). To analyze the potential influence of food television advertising on the eating behavior and nutritional status of children and adolescents.	A total of 116 students between 7 and 15 years old were selected to answer self-administered and self-explanatory questionnaires.	All children reported watching television daily. When asked about food advertisements on television, 46.9% reported liking and buying advertised food items and 54.9% said they were attracted to new products advertised through this medium. In addition, the study found a positive relation between the number of hours spent watching television and body weight.	Fair quality
Bacardí-Gascón et al.^34^ (2013)	Mexico, 2011	Quantitative study (cross-sectional). To determine the frequency of food advertisements on television targeting adults aired on two public access television channels and to explore the relation between foods advertised, foods consumed by mothers and children and the anthropometric data of both.	Two public access national television channels with the highest ratings were recorded during peak audience hours for a 5-month period. Women with children under 6 years of age from two states were recruited at educational and health facilities. They were asked to respond a self-administered questionnaire, attend an interview, and had their and their children’s weight and height measured.	A positive association was found between the frequency of television advertising and the weekly consumption of the foods advertised. A positive association was also found between hours spent by children watching TV and their BMI-Z scores.	Good quality
Chacon et al.^35^ (2013)	Guatemala, no year information.	Quantitative study (cross-sectional). To determine the availability of snacks that have child-oriented marketing strategies on the labels inside school stores and in stores surrounding schools; to assess their nutritional quality.	The availability of child-oriented snacks was assessed in stores inside schools and within a 200 square meters perimeter of 4 public schools in Mixco Guatemala. Nutritional quality was established following the UK Food Agency standards.	A total of 106 packages in 55 stores were analyzed. Savory snacks were the most commonly found child-oriented edible products. The most frequent marketing technique used was licensed characters (92.5%). Nutrition-related health claims were found in 41% of packages on child-oriented snacks. Most (97.1%) of the child-oriented snacks were classified as “less-healthy”.	Good quality
Díaz-Ramírez et al.^36^ (2013)	Mexico, 2011	Quantitative study (cross-sectional). To assess the frequency of food advertising broadcasted on 2 free-access television channels and to explore the associations between food advertisements and the eating patterns of mothers and their children.	Two free-access television channels with the highest ratings were recorded during weekdays at peak hours. A total of 365 mothers of children between 8 months to 5 years old and their children were interviewed to explore the type of foods consumed. The nutritional composition was assessed using the Mexican Health Bureau standards and the UK Food Agency standards.	Food products composed 25% of advertisements. Due to high levels of fat (25%), sugar (34%) and sodium (42%), 67% of them were classified as unhealthy. A positive correlation was observed between the food advertisements that mothers recalled and the frequency of advertisement. A positive correlation was found between the frequency of the foods advertised and their consumption by the mothers and their children.	Fair quality
Costa et al.^37^ (2013)	Brazil, 2009	Quantitative study (cross-sectional). To conduct a content analysis of television food advertising on broadcast channels during children’s programming hours.	Three public channels were recorded during weekdays and weekends. The categorization of foods was based on the Dietary Guidelines for the Brazilian Population.	Food advertisements represented 13.8% of all advertisements. The most frequent foods advertised were sugars and candies (48.1%) and oils, fats and oleaginous seeds (29.1%). By comparison only 1.1% of food advertisements promoted the consumption of vegetables and legumes.	Good quality
Rojas-Huayllani et al.^38^ (2013)	Perú, 2010	Quantitative study (cross-sectional). To study the influence of television advertisements on the consumption of unhealthy foods in elementary school children.	Children responded a questionnaire regarding television viewing habits. The most popular television shows were recorded and the food items advertised were presented to children in a second survey to determine whether they recognized the products and had consumed them.	From the students surveyed, 100% reported watching television for at least 3 hours every day. The foods advertised the most were cookies (31.5%), sweetened beverages (30.4%), chocolates (17.3%), and candies (9.2%). The consumption of food products that appeared on television was measured: Out of 120 students, 30 reported consuming 24 to 27 products, 23 students 21 to 23 products and 19 students 18 to 20 products. A positive association was found between unhealthy foods advertised on television and their consumption.	Fair quality
Busse et al^39^ (2014)	Perú, 2012	Qualitative study. To describe television viewing patterns and eating habits among elementary school children.	Data were collected from surveys, focus groups and in-depth interviews with children between 6 and 11 years of age and their parents.	Children watched between 5 and 7 hours of television per day and more during weekends. Focus groups showed that children recalled several television advertisements and request items advertised on television, especially toys and foods. Most boys and some girls were aware of the persuasive intentions of the advertisements.	Good quality
Gunderson et al^40^ (2014)	Honduras, 2012	Quantitative study (cross-sectional). To study the nutritional quality of foods and beverages advertised during children’s programming in Honduras and to describe the percentage of advertisements targeting children.	Four television stations were chosen; one public broadcast station and three cable stations. They were recorded and television commercials were coded and analyzed. Advertised foods were categorized as healthy and unhealthy, based on the classification scheme developed by Ramirez-Ley et al.	Foods and beverage advertisements accounted for 35.4% of all product advertisements in all channels. According to the food classification scheme, 69.8% of foods were unhealthy. Most of the advertisements for unhealthy foods were child-oriented (92.1%). In contrast to cable television channels, the public broadcast station did not air food advertisements aimed at children or marketing of unhealthy foods.	Good quality
Letona et al^41^ (2014)	Guatemala, 2013	Qualitative study. To determine the food products most frequently purchased by children, the reasons behind these choices and how children judge the nutritional value of food products.	Children between the ages of 7 and 12 years from two public elementary schools in Mixco, Guatemala, entered in focus groups where they participated in three separate activities: list making, picture selection and a drawing exercise.	The study included 37 children, who reported choosing foods based primarily on taste preference but also for its variety, large quantity and low price. Children perceived products with fruits and vegetables on the packaging to be healthy and some would not consume them. Children had literal interpretations of images and packaging, such as that Cheetos were made from cheese and Grape Soda from grapes.	Good quality
Mazzonetto et al^42^ (2014)	Brazil, 2014	Qualitative study. To analyze how children perceive their role in food purchases made by parents and the motivation behind their food preferences.	Focus groups were used to examine these topics with children between the ages of 8 and 10 years old that attend public schools in Florianópolis, Brazil.	Researchers found that children influence the food purchased in their houses and have the economic autonomy to make their own food purchases. Sensory appeal, taste and sight were the main motivations for their choices. Another stimulus reported was the television, both advertisements and food appearing in television programs. Energy-dense and nutrient-poor foods were associated with leisure activities such as going to the movies.	Fair quality
Mejía-Díaz et al^43^ (2014)	Colombia, 2012	Quantitative study (cross-sectional). To describe the nutritional profile of foods and nonalcoholic beverages advertised on television by type of audience (children’s programming vs. general programming).	Recording the two public access channels with the highest rating in Colombia, on weekdays and weekends. The foods were classified according to UK Food Standards Agency, WHO standards and Colombian micronutrient standards.	It was observed that 23% of advertisements were for food products. Out of these, 56% aired during children’s programming and 43.7% aired during general audience programming. Most foods and beverages advertised on children’s programming had high levels of sugar (69%) and sodium (56%) and medium to high levels of total fat (57.8%) and saturated fat (63.3%). None of the food products advertised during this programming were significant sources of fiber. In general programming, most foods and beverages were high in total fat (70.4%), and sodium (44%).	Good quality
Amanzadeh et al.^44^ (2015)	El Salvador, 2010	Qualitative study. To explore how highly processed foods and beverages are promoted in outdoor advertising in terms of methods and themes used by the advertisements and how it differs between rural and urban setting in El Salvador.	A photographic documentation of billboard and wall advertisements of foods was conducted. Field notes were collected during one week. Researchers identified patterns and explored the themes, topics and symbols revealed in the advertisements.	In total, 100 advertisements were recorded, 53 from rural areas and 47 from urban areas. The most frequent themes of advertisements were: “cheap price, large size and fast” found in more in rural than in urban advertisements, followed by “modern” also found in both but more in urban and then “refreshing” found in both settings.	Fair quality
Bridle-Fitzpatrick^45^ (2015)	México, 2011-2013	Mixed method study. To examine neighborhood environments (including outdoor advertising) in low-, middle-, and high-income communities in a Mexican city and to assess the foods available and the relationship that people develop with these foods.	Three schools representing communities of low, middle, and high socioeconomic status were selected in the city of Mazatlán during 2011-2013. Neighborhood food environments were assessed delineating three urban areas of approximately 1.5km^2^ that included schools and streets. Observational tools were used to assess the quantity, prices and promotion strategies of food and beverages products commonly consumed.	Lower-income and lower-middle-income families resided in areas with a greater number of traditional stores that gave them access to sweetened beverages and salty snacks. Upper-middle- and high-income areas have more large-scale supermarkets and fast food restaurants but less overall access to sweetened beverages and snacks. Almost all participants who resided in low- and middle-income areas included, in their photographic documentation, marketing strategies of different types for soft drinks and snack products. In high-income areas, only one student documented this kind of marketing.	Good quality
Cardenas et al.^46^ (2015)	Perú, no year information	Quantitative study (repeat cross-sectional study without control). To assess the effect of point of sale advertising of fruits in a University cafeteria in Lima, Peru.	Fruits were moved closer to the register desk, their price decreased, and signs were displayed to advertise their purchase.	Fruit sales showed a statistically significant increase between the initial state and the final stage of the study.	Fair quality
Chacon et al.^47^ (2015)	Guatemala, no information about the year it was conducted	Quantitative study (cross-sectional). To determine the most frequently marketed food to children in stores located inside and in the vicinity of schools and to assess the relations between food advertisement and proximity to schools.	Four public schools (two preschools and two primary institutions) were selected in the city of Mixco, Guatemala. Food stores located inside and within a 200-meter radius from these schools were surveyed to assess the number and type of foods and beverages advertised to children.	One third of food advertisements were child-oriented (29%). Stores located closer to schools had more display racks and shelves promoting child-oriented snack foods compared with those further away. The main items advertised were sweetened beverages (37%) and soft drinks (30%).	Fair quality
Ortiz-Pérez et al.^48^ (2015)	Mexico, 2012	Quantitative study (cross-sectional). To characterize the nutritional profile of processed foods advertised on Channel 5 of Mexican television.	Channel 5 was recorded during children’s programming hours for a week. Food advertisements were analyzed for duration and advertising strategy and the nutritional content was obtained from food packaging.	Food advertisements represented 36.4% of all advertisements. All foods displayed were processed foods with high levels of sugars and carbohydrates (74%), fats and sodium (17.5%), and milk products high in sugar (8.5%). No advertisements were found for unprocessed foods. The most advertised food group was sweetened cereals and was advertised using cartoons and had adventure and fun themes.	Fair quality
Soo et al.^49^ (2016)	Guatemala, 2013	Quantitative study (cross-sectional). To study the marketing strategies used to advertise breakfast cereals in Guatemala and to examine associations between several marketing strategies and nutritional quality.	All available breakfast cereals in Guatemala City were included in this study. A content analysis was performed to document child-oriented marketing, product claims, and health-evoking images. The Nutrient Profile Model (NPM) was used to calculate an overall nutrition score for each cereal (the higher the score, the lower the nutritional quality).	In total, 106 breakfast cereals were analyzed and half (50.9%) of them were child-oriented. The most common marketing strategy was the use of spokes-characters. More than half of the cereals (88.7%) contained a product claim on the package front such as “nutritious” and “whole grain” and almost all cereals (96.2%) had health evoking imagery. Child-oriented cereals had higher sugar content (10.1g versus 6.19g/30g) compared with non-child-oriented cereals. Cereals with health or nutrition claims were not significantly healthier than those without claims.	Good quality
Viacava et al.^50^ (2016)	Brazil, no year information	Mixed method study. To compare the content of advertisements used by alcohol, tobacco and food industries and to study similarities in strategy.	Images of tobacco, alcohol and food advertisements were found on Brazilian web pages and analyzed in terms of color, position, size and content analysis; to assess food nutritional facts, the criteria proposed by the Brazilian Health Regulatory Agency (ANVISA) were used to classify the products as “healthy” or “unhealthy”.	A total of 150 images were studied. Both the tobacco and alcohol industries use the color blue, whereas food advertisements use mostly red and green colors. These colors probably promote hunger and alter the perception that consumers have of the nutritional value of the product. More than half of the advertisements had the product name and brand occupying 0-25% of the advertising image (46%), followed by cartoons (17.3%) and celebrities (16.7%). The use of cartoons was most prevalent in the food products. More than half of the advertisements also used product appeal such as flavor, quality, and innovation. Another theme found was emotional appeal with themes such as physical attraction, happiness and sports. The nutritional assessment found that 82% of the foods advertised were unhealthy.	Fair quality
Britto et al.^51^ (2016)	Brazil, 2015	Quantitative (cross-sectional). To study child-oriented food advertisements aired on six Brazilian television channels and to describe the types of foods advertised and their nutritional content.	Six television channels in São Paulo with the highest ratings were recorded during several time periods, weekdays, and weekends. Food commercials were classified according to food type and nutritional value. The advertisements were analyzed to see if they met the National Council on the Rights of Children and Adolescents Resolution #163 of 2014 about food advertising.	Food and nonalcoholic beverage advertisements accounted for 5.6% of the advertisements. The most advertised foods on all six television channels were ultra-processed foods. Candy and packaged snacks were only advertised on one channel. Most of the food commercials (64.3%) used children’s language and characters; 43% used songs in children’s voices, and 21.4% used premium offers, against the National Council on the Rights of Children and Adolescents.	Fair quality
Busse.^52^ (2016)	Peru, 2002-2013	Quantitative (cross-sectional). To study and to describe the food advertisements that appear in child-oriented television programs in Peru and to study their effects on eating behaviors.	Twenty-five television programs, previously found to be children’s favorite, were recorded over a period of two weeks at three separate times of the year: spring, summer and winter.	Among the total number of advertisements recorded, 16.73% were food and beverage advertisements. In television programs, 28.3% included food items. Sweets and sweetened nonalcoholic beverages accounted for 47.6% of food and beverage advertisements. No advertisements aired fruits and vegetables. The most frequent marketing technique observed was the use of words to heighten the sensory appeal of foods (33%), followed by novelty (27%).	Fair quality
Castronuovo et al.^53^ (2016)	Argentina, 2015	Quantitative (cross-sectional). To analyze how mothers from Buenos Aires with different socioeconomic levels perceive food advertisements.	Eight focus groups were conducted with the participation of 49 mothers with different education levels and who had children between the ages of 5 and 13. A questionnaire was conducted in the focus groups aiming to discuss the importance of food advertising at the moment of making decisions about their children’s diet and health.	Researchers found children influence the purchasing decisions of their mothers, and mothers perceive that television advertising by spokes-characters and premium offers impel children to crave certain foods and beverages. Mothers also reported being impelled to buy certain food for their children based on nutritional or health claims. Mothers said that both them and their children were susceptible to brand loyalty.	Fair quality
Mazariegos et al.^54^ (2016)	Guatemala, no year information	Quantitative (cross-sectional). To describe and assess the use of toys and price incentives in fast food chain restaurants in Guatemala and to explore the nutritional quality of children’s combo-meals with health claims.	Children’s combo-meals from all major fast food chains in Guatemala were purchased and their marketing strategies were analyzed. Nutritional profiles were assessed based on the UK Nutrient Profiling Model.	In total, 114 children’s combo-meals were found with a frequency of 9.5% to 25% per fast food restaurant. All these combo-meals included a toy giveaway. Nutritional information was only available for 2 of the 6 restaurants. All the children’s combo meals were “less healthy” according to the NPM even though three of these meals had health claims.	Good quality
Patiño et al.^55^ (2016)	Mexico, 2012-2013	Quantitative (cross-sectional). To assess the nutritional quality of foods advertised on Mexican television according to the Mexican nutritional standards, World Health Organization standards and the European and United Kingdom Nutrient Profiling Model (UKNPM).	Four broadcast stations with the highest national ratings were recorded, including broadcast for several audiences and types of programs. Advertisements were classified and the nutritional profiles of the food items were obtained. Three nutritional models were used to assess the nutritional quality of the food items.	Food and nonalcoholic beverage advertisements represented 20.7% of all advertisements recorded. Those that aired during cartoon programs had the highest energy and sugar content and had the lowest nutritional quality when compared with other types of programming. More than 60% of foods advertised did not meet any standard nutrient profile model; 64.3% did not meet the Mexican nutritional standards, 83.1% did not meet the WHO standards and 78.7% did not meet UKNPM standards. During cartoon programming only 33.9%, 8.6% and 17.2% of the foods complied with the Mexican, WHO Europe and UKNPM standards, respectively.	Good quality
Théodore et al.^56^ (2016)	Mexico, no information about the year it was conducted	Quantitative (cross-sectional). To assess child-oriented television advertisements by companies who have signed self-regulation agreements.	Broadcast television channels with the highest national ratings were recorded and the commercials were classified and analyzed.	More than half (74.9%) of all advertisements tried to influence children’s purchases and consumption. Companies that signed self-regulation agreements and those that did not sign advertised mainly unhealthy foods and beverages. Companies that signed self-regulation agreements had less advertisements directly aimed at children.	Good quality
Zucchi et al.^57^ (2016)	Brazil, no year information	Mixed methods study. To study and describe the nutrient claims printed on food packaging of child-oriented ultra-processed foods and explore children’s understanding of these marketing strategies.	A supermarket belonging to one of the largest supermarket chains in Brazil was chosen for this study. Packaged foods were identified and classified according to their target audience. Nutritional information available on the packaging was used to determine which foods were ultra-processed. These ultra-processed foods were then classified according to their nutrient claims. Focus groups were conducted with children between 8 to 10 years old to explore their perception of package advertising on ultra-processed foods.	The researchers identified 535 packaged foods that were child-oriented. Among these, 472 or 88% were classified as ultra-processed foods. Within this group of ultra-processed foods, 46.6% had at least one nutrient claim on the packaging front. The most common nutrient claims were the presence of vitamins and minerals followed by reduction claims regarding trans fats. Nine focus groups were carried out with 49 children. They showed interest in spokes-characters and reported liking the fact that foods were marketed directly at them as an age group. They considered nutrient claims were directed at parents. Their interpretation of health claims was considered a positive characteristic of the food product.	Fair quality
Rovirosa et al.^58^ (2017)	Argentina 2013-2014	Quantitative (cross-sectional). To study and describe the frequency and duration of food and beverage advertisements aired during children’s programming, and the nutritional quality of these foods.	Children’s programming aired in the city of Buenos Aires was recorded during weekdays and weekends. Television advertisements were analyzed, and the nutritional data of the products was assessed using the Nutrient Profiling Model and the traffic light labeling system.	Food and beverage advertisements represented 20.9% of the television advertisements recorded. Dairy products, candies, and fast-food meals were the most advertised food products. According to the Nutrient Profiling Model only a third of advertised foods and beverages were healthy. Based on the traffic light labeling system almost half of the food products was high in sugar, a quarter was high in saturated fats, and 15% was high in sodium and total fats.	Fair quality
Allemandi et al.^59^ (2017)	Argentina 2013-2014	Quantitative (cross-sectional). To characterize the television advertisements of ultra-processed foods targeted to children.	The three most popular cable channels targeted at children and five broadcast channels were recorded for 6 weeks. Foods were classified following the NOVA system. PAHO nutrient profile model was used to assess the nutritional quality.	Ultra-processed foods were advertised the most during programs aimed at children (98.9%), compared with programs targeted towards general audience (93.7).	Good quality

Appendix II shows the results of the quality assessment, in which zero studies were judged as being poor quality, 21 had fair quality and 16 had good quality.

**Figure 2:**
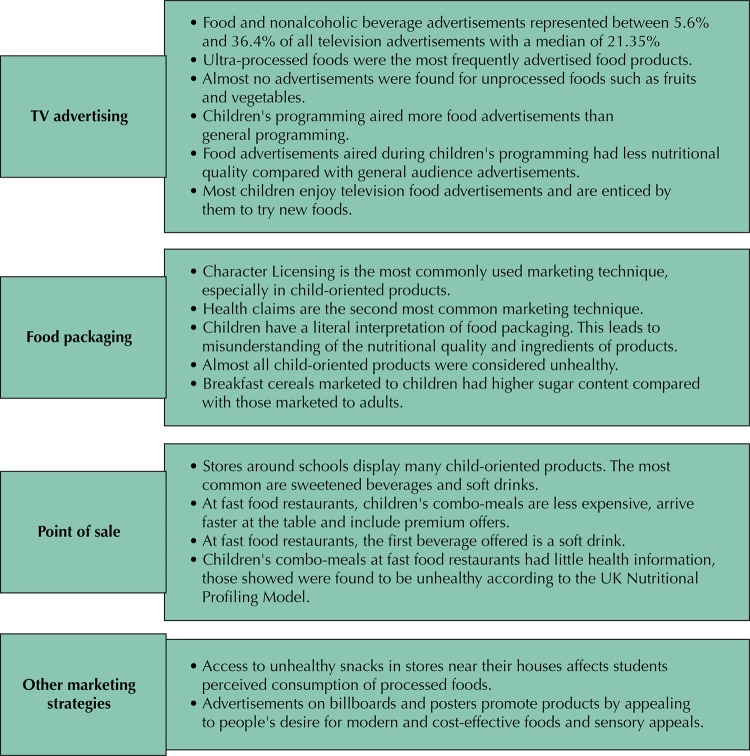
Main findings based on marketing typology.

The thematic analysis identified the following typologies: a) television advertisement, b) food packaging marketing, c) marketing strategies at points of sale and d) other marketing strategies. Figure 2 shows the main findings of the review based on these typologies.

Twenty-five studies inquired several topics related with television advertisement. Fourteen of them found that food and nonalcoholic beverage advertisements represented between 5.6% and 36.4% of all television ads^24,27-29,34,36,37,40,43,49,52,53,56,59^.

Seventeen studies found the most frequently advertised food products were ultra-processed foods, whereas almost no advertisements were found for unprocessed foods such as fruits and vegetables^24,27,29,31,34,36-38,40,43,49,52,53,56,57,59,60^

Several studies also found that more food advertisements appear during children’s programming compared with general audience programming and that child-oriented advertisements had less nutritional quality compared with general audience advertisements^24,28,40,43,52,56,57^.

Ten studies found most children enjoyed television food advertisements and are enticed by these ads to try new foods^25,26,30,32-34,38,39,42,54^.

A Mexican study assessed the self-regulation agreement implemented by the industry in that country in 2012. The authors found that 93% of the advertisements, from companies that had signed the self-regulation agreements, promoted unhealthy food and beverages^57^.

Three studies found the most common marketing technique used on food packages was licensed characters — especially in child-oriented products — followed by the presence of health claims^35,50,58^.

A Guatemalan study found children had a literal interpretation of food packaging, which leads to a misunderstanding about the nutritional value and the ingredients of products; children believe, for example, that the food drawn on the packaging represents the ingredient of the product inside.^41^ Another study, which assessed snack foods available in stores near schools, found that almost all child-oriented products were considered unhealthy according to the UK Food Agency standards.^35^ A third study, which gathered breakfast cereals available in supermarkets in Guatemala City, observed cereals marketed to children had higher sugar content compared with cereals marketed to other age groups.^50^

Another study conducted in Guatemala analyzed the point of sale marketing strategies in stores located around schools to determine the relation between food advertising and proximity to schools. This study found many child-oriented products with a proportional relation between them and the distance to the school. The most common products found were sweetened beverages and soft drinks^48^.

Also in Guatemala, a study exploring point of sale marketing in fast food restaurants observed that only five children’s combo meals out of 19 had nutrition information, and all of them were classified as “less healthy” according to the UK Nutrient Profiling Model. On average, combo meals were less expensive than children’s meal items individually. All restaurants offered a soft drink as the first drink option and all combo meals included a toy giveaway^55^.

Advertisements on billboards and posters promote products by appealing to people’s desire for modern and cost-effective foods vs traditional foods^45^.

Access to unhealthy snacks in stores near students’ houses affects their perceptions of consumption of processed foods. Students who were asked to record the food they had access to, observing that, if unhealthy foods were available, they felt tempted to consume them^46^.

## DISCUSSION

This scoping review synthesizes the evidence about the marketing of foods and beverages in Latin American countries in the last 15 years and provides insights about the challenges this issue represents to global and public health. This study also shows the different ways in which marketing companies interact with children when promoting ultra-processed foods and beverages in this region. Finally, it allows the identification of knowledge gaps that can guide the future research agenda in this area.

The main findings of this review can be described as follows: a) most foods and beverages advertised in television are ultra-processed foods and have low nutritional value^24,27,29,31,34,36-38,40,43,49,52,53,56,57,59^; b) children’s television programming contains several food advertisements and many studies found advertisements for this demographic group had a lower nutritional value compared with general audience programming^24,28,40,43,52,56,57^; c) food packages can be deceiving especially to children who have a literal interpretation of what is printed on the packaging^35,50,58^; d) proximity to unhealthy foods may increase the consumption of these foods^25,26,30,32,34,38,42,44,46,54^. These findings enhance the necessity of implementing statutory policies covering all marketing channels and robust nutritional standards as the case of the Chilean regulations approved in 2012^60^. Similarly, the Brazilian food dietary guidelines and the Uruguayan food guidelines emphasized on encouraging and increasing consumption of natural and minimally processed foods while making recommendations for marketing^61,62^.

Television is one of the main channels used to advertise food products^63^ and more than half of the 37 studies included in this review focused on television advertising. The frequency of food advertisements was between 5.6% and 36.4% with a median of 21.35%^24,27-29,34,36,37,40,43,49,52,53,56^. These results agree with those found by other studies worldwide. In the United States, a study by Powell et al^64^ found 36.4% of television advertising corresponded to food promotions, a study also by Powell^65^ comparing television food advertisements seen by children in 2003 and 2007 saw a slight reduction from 13.1%-13.6% of ads watched by children in 2003 to 11.5%-13.6% in 2007. In the United Kingdom, Whalen et al.^66^ studied food promotions aired on television after statutory restrictions; food and drink ads were the third most advertised product with a frequency of 11.9%. In China, a recent study by Danyong et al.^67^ reports that food advertisements constitute 25.5% of television ads and in a comparative study conducted in 2010 by research groups in Australia, Asia, Europe, North America and South America, food items represented 18% of all television advertisements and represented the second most advertised product^17^.

The most frequently advertised foods on television were ultra-processed foods and sweetened beverages, with few advertisements for healthier options such as fruits and vegetables^24,27,29,31,34,36-38,40,43,49,52,53,56,57,59^. Researchers in developed countries have been repeatedly making this observation ^65,68^. For example, in 2007, Powel et al.^69^ found almost all food products advertised to children and adolescents in US television were high in fat, sugar or sodium. Another study also conducted in the US observed foods advertised on television contained in general too much sugar, fat and protein and too few servings of dairy, fruits and vegetables^70^.

Several of the studies included in this review also observed that children were heavily targeted by marketing and that foods marketed to them had a lower nutritional value compared with food advertised to adults^24,28,40,43,52,56,57^. In 2008, WHO published a review of the evidence regarding food marketing to children, including 14 studies that compared television advertising to both populations, which found a larger proportion of food advertisements for children vs. adults^63^.

This is especially troubling since children’s exposure to marketing of unhealthy foods has been connected to an increased energy intake and childhood obesity^71,72^. Latin America is experiencing a rapid increase in obesity and other associated non-communicable diseases in children and young adults^73^, and in 2014 a systematic review by Rivera et al. found that from 20 to 25% of the population were already obese^74^. We still have no knowledge of a study that analyzes the relation between television food advertising and childhood obesity rates in Latin America. According to a recent international comparison, the magnitude of the television advertising effect on overweight and obesity in children varies by country^75^, highlighting the necessity for evidence-based studies in Latin America illustrating this effect.

While numerous studies have focused on the nature of the food advertised on television, little can be said about the nature of other above-the-line advertising such as printed media. We found only one study that investigated advertisements printed on billboards, which were found to be all for fast food chains^45^.

This review did not find studies about the use of digital marketing. Adults and children across the world have increasing access to digital media, especially with the widespread availability of cellphones and, as such, digital marketing is becoming more significant^76,77^; it allows brands to emit widely amplifying advertising messages, achieving high levels of recall and brand awareness^77^. Additionally, digital marketing benefits from the personal information collected about the user and how this fact might affect consumers is still unknown. Latin America is a growing market for digital advertising with more than half of its population connected to the Internet, and as of 2015, it is the fourth largest mobile device market in the world^78^. As youth become increasingly and permanently connected through mobile media, new research in this region is needed to understand the prevalence and effects of digital marketing on children’s health and consumption behaviors.

Below-the-line promotional techniques such as product packaging and product placement inside stores, as well as sponsorship of food items in movies or television programs, toy giveaways, free samples, and loyalty programs are also under-researched. We found nine studies that focused on food packaging and point of sale advertising. The results are similar to those of television advertising with most of the child-oriented products being ultra-processed foods and sweetened beverages. Studies found that children have a literal interpretation of food packaging and tend to believe the images and health claims printed on the packages^41,50^. Children also reported that easy access to unhealthy foods such as having stores inside and around schools and in the vicinity of their houses affected their consumption^46^. These findings place children in a particularly vulnerable situation when confronting purchasing decisions made with pocket money and are the development to an unhealthy diet in adult life.

Therefore, new research examining the effects of external factors (amplifying and attenuating) such as school environment, family communication styles and parental advertising mediation is needed. Family communication styles and parental advertising mediation has been found to moderate the effect of television ads geared towards children^79^, including food television advertising^80^. Other studies have analyzed how cognitive development and other psychological factors help children cope with advertising messages. Findings in these studies suggest children’s executive function and advertising literacy directly affect children’s consumer behavior when exposed to advertising^81,82^ and can help to moderate the effect.

Several limitations can be identified in this review. Firstly, despite the exhaustive search used in this review, we have the probability of having excluded thesis or dissertations conducted in the area that were not published in indexed journals. Secondly, we did not find studies conducted in countries such as Venezuela, Bolivia, Paraguay, and Panama, which limits the reach of conclusions of this review. Thirdly, the exploratory scope of this review does not enable conclusions and recommendations about specific topics of food marketing to be made. In consequence, this study should be considered as a preliminary approximation to this topic.

Despite these limitations, the results of this study may provide insights to guide policy actions to prevent the growing public health problem of obesity and non-communicable diseases. Furthermore, it can contribute to identifying knowledge gaps to lead the future research agenda in the area.

## CONCLUSIONS

This review synthesizes the evidence published in the last 15 years on marketing of food and beverages in Latin American countries. The main advertising medium explored by these studies was television advertising, and while studies focusing on digital advertising were not found, it is also gaining strength, especially among youths. These media transcend national borders and, as such, it is necessary to view the panorama of the region as a whole. This review provides insights about the challenges unhealthy eating represents to the public health of Latin America and identifies knowledge gaps to guide future research.
