# Erratum

**DOI:** 10.11606/s1518-8787.2019053001184err

**Published:** 2020-05-15

In the article: **“Scoping review of studies on food marketing in Latin America: Summary of existing evidence and research gaps”** Rev. Saude Publica [online]. 2019, vol.53:107, ISSN 1518-8787, http://dx.doi.org/10.11606/s1518-8787.2019053001184, the RSP corrects the publication type and the How to cite:

**Where you read:**

**Revisão**

**How to cite:** Chemas-Velez MM, Gomez LF, Velasquez A, Mora PLazas MM, Parra DC. Scoping review of studies on food marketing in Latin America: Summary of existing evidence and research gaps. Rev Saude Publica. 2018;53:107.

**You should read:**

**Review**

**How to cite:** Chemas-Velez MM, Gomez LF, Velasquez A, Mora PLazas MM, Parra DC. Scoping review of studies on food marketing in Latin America: Summary of existing evidence and research gaps. Rev Saude Publica. 2019;53:107.

